# Biocompatible 3D-Printed Devices With Adipose Stem Cells in the Regenerative Process of Sciatic Nerve Lesions in Rodent Models: An Experimental Study

**DOI:** 10.7759/cureus.62412

**Published:** 2024-06-14

**Authors:** Cristian Trambitas, Timea Pap, Raluca Niculescu, Maria Catalina Popelea, Ovidiu S Cotoi, Bogdan Cordoș, Horatiu-Paul Domnariu, Andrei Marin, Andrei Marian Feier, Camelia David, Cristian Vintila

**Affiliations:** 1 Plastic and Reconstructive Surgery, George Emil Palade University of Medicine, Pharmacy, Science, and Technology of Targu Mures, Targu Mures, ROU; 2 Pathology and Laboratory Medicine, George Emil Palade University of Medicine, Pharmacy, Science, and Technology of Targu Mures, Targu Mures, ROU; 3 Physiopathology, George Emil Palade University of Medicine, Pharmacy, Science, and Technology of Targu Mures, Targu Mures, ROU; 4 Center of Experimental and Imaging Studies, George Emil Palade University of Medicine, Pharmacy, Science, and Technology of Targu Mures, Targu Mures, ROU; 5 Plastic and Reconstructive Surgery, University of Oradea, Lucian Blaga University of Sibiu, Oradea, ROU; 6 Plastic and Reconstructive Surgery, Carol Davila University of Medicine and Pharmacy, Bucuresti, ROU; 7 Orthopaedics, George Emil Palade University of Medicine, Pharmacy, Science, and Technology of Targu Mures, Targu Mures, ROU; 8 Plastic and Reconstructive Surgery, Emergency County Hospital Targu Mures, Targu Mures, ROU

**Keywords:** 3d-printed guide, experimental sciatic nerve defect, muscle atrophy, adipose stem cells, nerve regeneration

## Abstract

Introduction: Peripheral nerve injuries are a significant clinical challenge. The rat sciatic nerve serves as an ideal model for studying nerve regeneration. Extensive research has been conducted to unravel the intricate mechanisms involved in peripheral nerve regeneration, aiming to develop effective therapeutic strategies for nerve injury patients. Research including different types of materials that can be used as nerve guides like synthetic polymers have been investigated for their biocompatibility and molding properties. Among multiple stem cell types, adipose-derived stem cells (ASCs), bone marrow-derived mesenchymal stem cells (BM-MSCs), and induced pluripotent stem cells (iPSCs) have shown neuroprotective and regenerative important properties.

Methods: The purposes of our study were to develop a protocol for rat sciatic nerve injury treated with 3D-printed guide and adipose stem cells to investigate nerve regeneration through histologic examination and biomechanical characteristics of muscular tissue. We use 20 (100%) male Wistar rats, measuring between 350 g ± 35 g, who underwent complete transection of the right sciatic nerve, resulting in a 1 cm defect. The group was separated into three subgroups: the first subgroup (n = 8) was treated with a 3D-printed guide with adipose stem cells, the second subgroup (n = 8) was treated with a 3D-printed guide without adipose stem cells, and the third subgroup (n = 4) was the control group. At four, eight, and 12 weeks, we measured with ultrasonography the grade of muscular atrophy. At 12 weeks, we harvested the sciatic nerve and performed a histological examination and mechanical investigation of the tibialis anterior muscle.

Results: On the examined specimen of the first subgroup, cross-sectioned nerve structures were present, surrounded by a mature fibro-adipose connective tissue, with blood vessels. In the second subgroup, no nerve structure was observed on the examined sections, but in the polymorphic inflammatory infiltrate and control group, no signs of regeneration were found.

Conclusions: The present study shows a promising potential when utilizing adipose stem cell-based therapies for promoting peripheral nerve regeneration following large (>1 cm) nerve defects knowing that at this size, regeneration is impossible with known treatments.

## Introduction

Peripheral nerve injuries are a significant clinical challenge, often resulting in loss of motor function, sensory deficits, and impaired quality of life [1,2]. Nerve transection, characterized by complete severance of the nerve fibers, poses a particularly daunting hurdle for successful nerve regeneration. The sciatic nerve, a critical component of the peripheral nervous system, serves as an ideal model for studying nerve regeneration due to its accessibility and functional importance in motor and sensory innervation of the lower limb. Numerous structural and functional alterations take place in cases of peripheral nerve injuries. The sensory and motor functions experience a decline despite optimal surgical repair of peripheral nerves. This decline resulting from peripheral nerve injuries correlates with the nature of the injury and the chosen repair technique. Oxidative stress poses a threat to peripheral nerve regeneration and the repair process following nerve lacerations [3].

Over the years, extensive research has been conducted to unravel the intricate mechanisms involved in peripheral nerve regeneration, aiming to develop effective therapeutic strategies for nerve injury patients. Animal models, particularly rats, have played a crucial role in elucidating the complex biological processes underlying nerve regeneration and evaluating potential interventions [4-7].

The objective of this study is to investigate the regenerative capacity of the peripheral nervous system by examining the process of nerve regeneration in rats following complete transections of the sciatic nerve. By understanding the cellular events involved in this regenerative process, we aim to contribute to the development of novel therapeutic approaches that can enhance functional recovery after peripheral nerve injury [8,9].

## Materials and methods

The specimens used were provided by the animal facility of George Emil Palade University of Medicine, Pharmacy, Sciences and Technology (UMFST) of Targu Mures, Romania, and were housed in standard laboratory conditions with a 12-hour light/dark cycle, a temperature of 22 °C ± 2 °C, and a humidity of 60 ± 5%. The rats were kept in singular cages and provided standard pellet feed and water ad libitum. Animal experimental procedures performed in this study were according to the guidelines of the Institutional Animal Care and Use Committee, approved by the Ethical Committee of George Emil Palade UMFST of Targu Mures.

**Figure 1:**
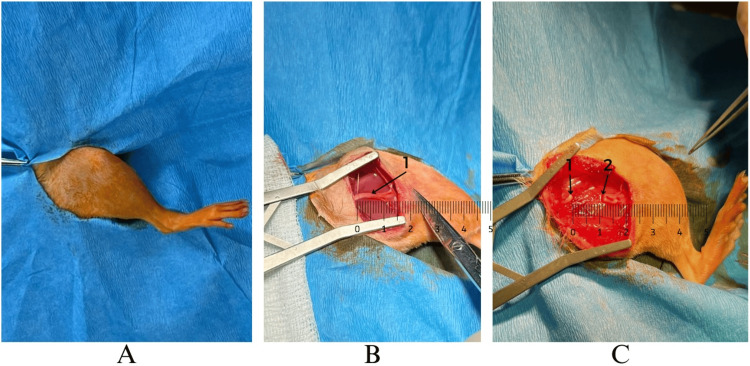
Wistar rat under anesthesia and the dissection maneuver, identifying the peripheral sciatic nerve. A: right inferior limb of the rat isolated; B: exposer of the sciatic nerve, B1: sciatic nerve; C: gap between two ends of the sciatic nerve, C1: proximal stump of the sciatic nerve, C2: distal stump of the sciatic nerve

We employed a total of 20 male Wistar rats, aged between 10 and 12 months and weighing between 350g ± 35 g, for our experimental procedures. The rats underwent complete transection of the sciatic nerve, resulting in a 1 cm defect. Out of the total, 16 rats were allocated to the study group, while the remaining four rats formed the control group. The control group underwent the same surgical procedure but did not receive any nerve repair, serving as a reference (Figure 1).

All surgical procedures were conducted under continuous general anesthesia. The rats were initially placed in an induction chamber with a flow rate of 0.8 L/minute of O_2_ and 5% isoflurane. Subsequently, during the surgical procedure, inhalation anesthesia was administered via a mask with a flow rate of 0.6 L/minute of O_2_ and 2-3% isoflurane.

**Figure 2:**
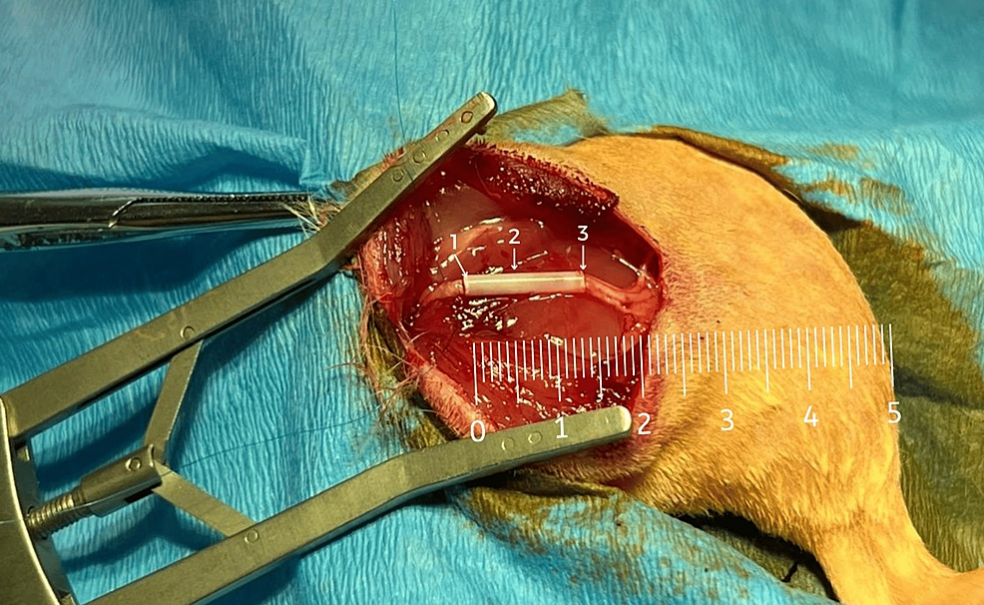
The 1 cm sciatic nerve defect with the interposed 3D-printed guide. A) with adipose tissue stem cells. 1: proximal stump of the sciatic nerve inserted in the 3D-printed guide, 2: the 3D-printed guide, 3: distal stump of the sciatic nerve inserted in the 3D-printed guide

The study group was subdivided into two distinct subgroups (Figure 2): The first subgroup (n = 8) received a 3D-printed tube made of polylactic acid (PLA) filled with adipose stem cells, which was interposed at the site of the defect. The second subgroup (n = 8) was treated with 3D-printed tubes without stem cells.

At the end of the 12-week experimental period, the animals were humanely euthanized by administering an overdose of isoflurane, and the tubes along with the distal and proximal ends of the nerves were dissected, harvested, and sent to the laboratory for histopathological analysis. The obtained probes were stained with hematoxylin and eosin (H&E) and S100 immunoreaction.

Computer-aided design (CAD) software (SolidWorks, Dassault Systèmes, France) was used in designing the 3D nerve guide, and for printing, we used a fused deposition modeling (FDM) 3D printer (MakerBot Replicator, MakerBot Industries, New York, NY, USA) with a polylactic acid (PLA) filament. The nozzle temperature used for printing was set at 200°C and bed temperature at 60°C.

The stem cells were obtained from the interscapular fat pad. An incision to the dorsal area was made and careful dissection until the fat tissue was observed. A piece of the fat tissue was harvested of approximately 1 cm^3^ and introduced in a tube with a sterile growth factor inside (50 ml DMEM5 + 10% FBS6 + 2% antibiotic/antifungal) and transported to the tissue engineering lab within 30 minutes. 

The author used this protocol for obtaining and multiplying the stem cells in other previous studies [10,11]. The harvested tissue was shredded with a sterile scalpel and scissors into pieces as small as possible (1 x 1 mm). The tissue was immersed in type 1 collagenase solution and incubated for one hour in a water bath to detach the stem cells from the adipose tissue. Filtration was done to remove residual fatty tissues and centrifugation for five minutes at 5000 rpm to remove the collagenase by suction, followed by immersion in ammonium chloride to destroy the red blood cells. The process continued with centrifugation for five minutes at 5000 rpm to remove ammonium chloride by suction. The collection of cells was placed in a culture medium preheated to 37°C and placed in flasks in the incubator at 37°C and 5% CO^2^ environment.

## Results

**Figure 3:**
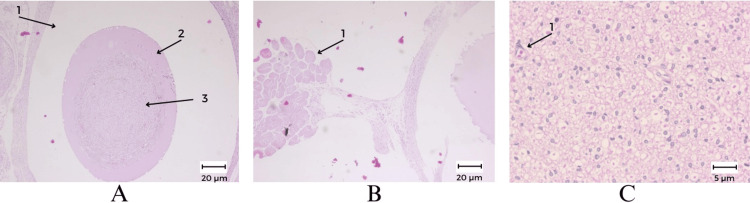
Histological results showing the 3D-printed tube filled with the cross-sectioned nerve structure, surrounded by the perinervous tissue. A: Nerve bundle surrounded by a space suggesting the 3D printed guide (hematoxylin and eosin, 10x), A1: former 3D-printed guide, A2: marked edema and amorphous material, A3: nervous structure. B: Striat muscle surrounding  the 3D-printed guide (hematoxylin and eosin, 10x), B1: striated muscle. C: nervous structure (hematoxylin and eosin, 40x), C1: vascular structure.

On the examined sections of the 3D-printed tube + stem cells, cross-sectioned nerve structures were observed, consisting of nerve bundles, surrounded by a mature fibro-adipose connective tissue, with blood vessels (very similar to the peri-nervous tissue). Each nerve bundle has a normal histological structure, made up of myelinated nerve fibers, with blood capillaries among them. In the middle of the section, a space was observed (suggesting the PLA printed tube) surrounding one of the previously described bundles showing edema at the level of the perinerve, inside which marked edema and an amorphous material was observed, eosinophilic, with the presence of some inflammatory cells (lymphocytes and eosinophils) (Figure 3).

**Figure 4:**
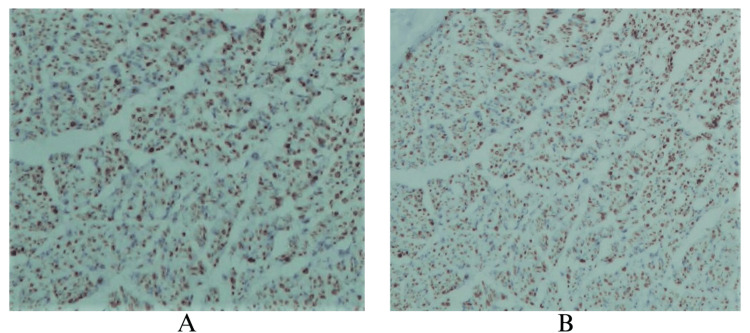
Nervous structure inside the 3D-printed guide, positive to nuclear S100 imunohistochemistry marker, confirming the presence of neural tissue A: Nervous structure inside the 3D-printed guide positive to S100, 40x. B: Nervous structure inside the 3D-printed guide positive to S100, 20x.

Immunohistochemically, the anti-S100 antibody labels nerve cells and adipocytes (Figure 4).

In the second group, no nerve was observed on the examined sections, but polymorphic inflammatory infiltrate, predominantly composed of granulocytes. Multinucleated foreign body-type giant cells and macrophages loaded with brown pigment (hemosiderin) were also seen.

There were no signs of regeneration in the proximal or distal sciatic nerve stump of the control group (Table 1).

**Table 1:** Histological and immunohistochemical results for the three subgroups examined at 12 weeks after surgery.

Group (n = 20)	Applied treatment	Histological results	Immunohistochemical
First subgroup (n = 8)	PLA 3D-printed guides + stem cells	Cross-sectioned nerve structures with nerve bundles surrounded by fibro-adipose tissue and blood vessels.	Nerve cells and adipocytes
Second subgroup (n = 8)	PLA 3D-printed guides	No nerve on the examined sections, only polymorphic inflammatory infiltrate, predominantly composed of granulocytes	No examination
Control subgroup (n = 4)	No treatment	No nerve on the examined sections	No examination

## Discussion

Following peripheral nerve injury, there exists a certain degree of inherent capacity for repair and regeneration [12,13]. Extensive scientific investigations have been undertaken to comprehend the underlying mechanisms and devise strategies aimed at enhancing this regenerative process. Nevertheless, recuperation from severe nerve transections often poses substantial challenges, and the outcomes achieved thus far remain less than optimal [14-17].

Histologically, the peripheral nerves have two major components: stroma and parenchyma. The stroma consists of three layers of connective tissues, while the parenchyma is formed by the nerve fibers, namely, axons and Schwann cells. The nerve fibers can be myelinated if the Schwann cells cover a single axon, or a single Schwann cell can interact with a group of axons resulting unmyelinated nerve fibers [18-23].

This work has demonstrated that in vivo nerve regeneration is possible through a PLA 3D-printed tube filled with adipose-derived stem cells (ASCs) from a histological point of view. The examination of the second group showed a significant difference with no neural growth inside the tube, suggesting that a key role in nerve regeneration on large defects is played by the scaffold material associated with the stem cells.

Duvernay published a paper after studying chitosan conduits with and without ASCs for nerve regeneration showing favorable clinical, histological, and functional results in favor of conducts with ASCs and then chitosan conducts alone [24].

ASCs encapsulated in a laminin-peptide-functionalized hydrogel (Biogelx-IKVAV) within a poly-ε-caprolactone nerve conduit promoted robust axonal elongation and Schwann cell proliferation across a 15 mm critical-size gap in a rat sciatic nerve defect, suggesting their potential in improving nerve regeneration [25].

Other studies used 3D printing technology to improve results and obtain better outcomes for nerve regeneration, which seems to be a good pathway for further investigations [26,27].

We decided on the three-month experimental period because Hansen [28] demonstrated that the healing process had plateaued after this period in his experiment when he analyzed the locomotion of the rats at three and six months postoperatively. However further research should be done in order to histologically investigate and understand the nerve growth, scarring, and tissue remodeling at longer periods of time and correlate with the clinical findings. 

Nerve transection initiates the degenerative process in the distal portion of the nerve, known as Wallerian degeneration (WD). Schwann cells play a crucial role during WD, responding to the loss of axons by actively extruding their myelin sheaths, downregulating the expression of myelin genes, undergoing dedifferentiation, and engaging in cellular proliferation. Subsequently, they begin expressing surface molecules that facilitate the guidance of regenerating nerve fibers [23,27,29].

In addition, hematogenous macrophages are swiftly recruited to the distal nerve stump, where they undertake the essential task of clearing most myelin debris. Molecular alterations within the distal stump encompass the upregulation of neurotrophins, cell adhesion molecules, cytokines, and their corresponding receptors. These molecular changes contribute to the complex and intricate process of nerve regeneration [30-32].

Following nerve injury, hypoxia triggers angiogenesis through vascular endothelial growth factor, leading to the expansion of existing vessels into new regions. Manipulating specific signaling pathways in stem cells can enhance angiogenesis by releasing different angiogenic factors. In addition, stem cells play a critical role in peripheral nerve regeneration by promoting myelin production and being responsive to environmental cues that drive their differentiation into Schwann-like cells [15].

Blood vessels form tracks for Schwann cells to migrate and guide axonal growth before axonal extension during regeneration, indicating a close relationship between neurite outgrowth and vascularity [33,34].

Other studies have shown that nerve gaps over 1 cm in rats are unlikely to regenerate even with surgical repair [14,16] if the space in between the nerve stumps is not filled with supportive cells (Schwann cells) to provide a growth environment for the axon [33-36]. In our experiment, we also provided an easy-to-access source of neural progenitor cells derived from the adipose stem cells with great histological results in nerve regeneration.

It is important to discuss the limitations of our study. We did not perform a histological analysis of the muscle samples to investigate muscular atrophy. Our study utilized only a concentration of ASCs (2 × 10^6^). To enhance our research, we intend, in the future, to use higher concentrations to ascertain more potential benefits. Our will is to expand this study by analyzing the structural remodeling of muscle tissue, monitoring muscle evolution, and analyzing the mechanical behavior of the contralateral muscle.

## Conclusions

Our study highlights the promising potential of utilizing stem cell-based therapies for promoting nerve regeneration following peripheral nerve injuries. Histological and immunohistochemically analysis revealed notable advancements in the regenerative process within the study group, characterized by the presence of cross-sectioned nerve structures, myelinated nerve fibers, and a marked reduction in inflammatory infiltrates compared to the control group. The presence of neural structures inside the guide with ACS corroborates with muscle recovery and demonstrates nerve regeneration in our model.
